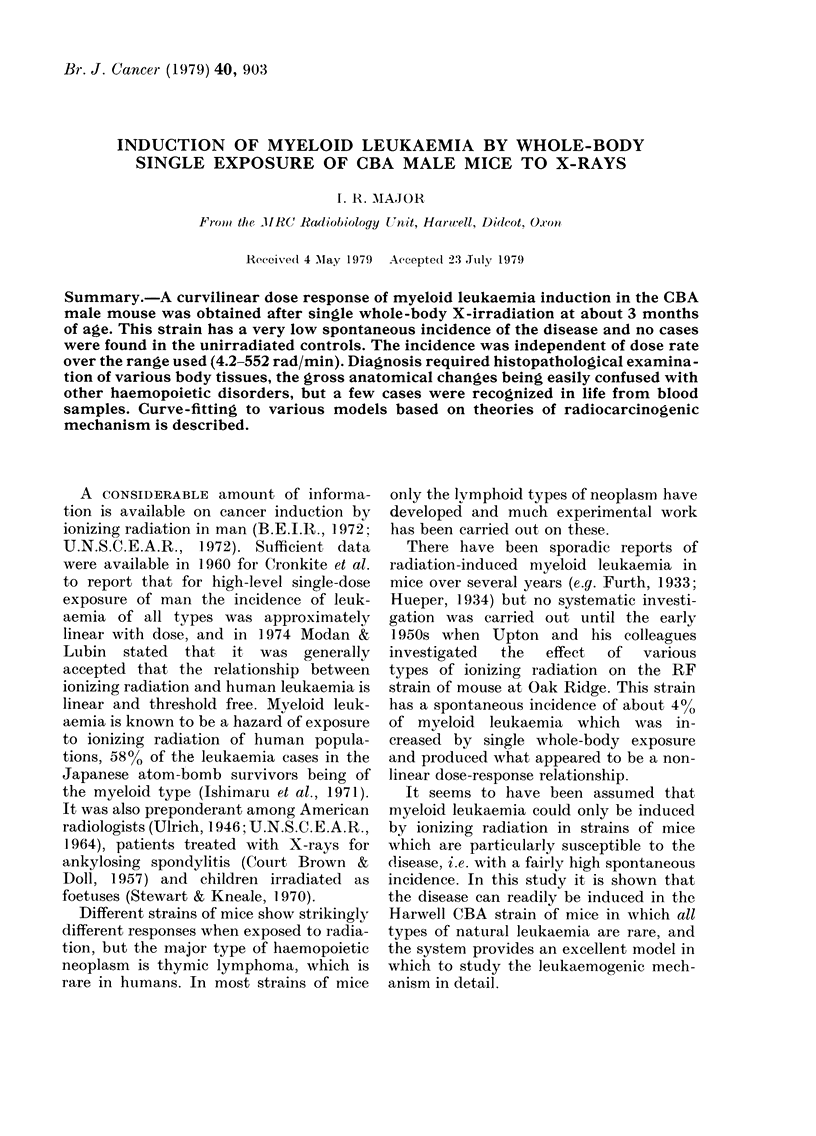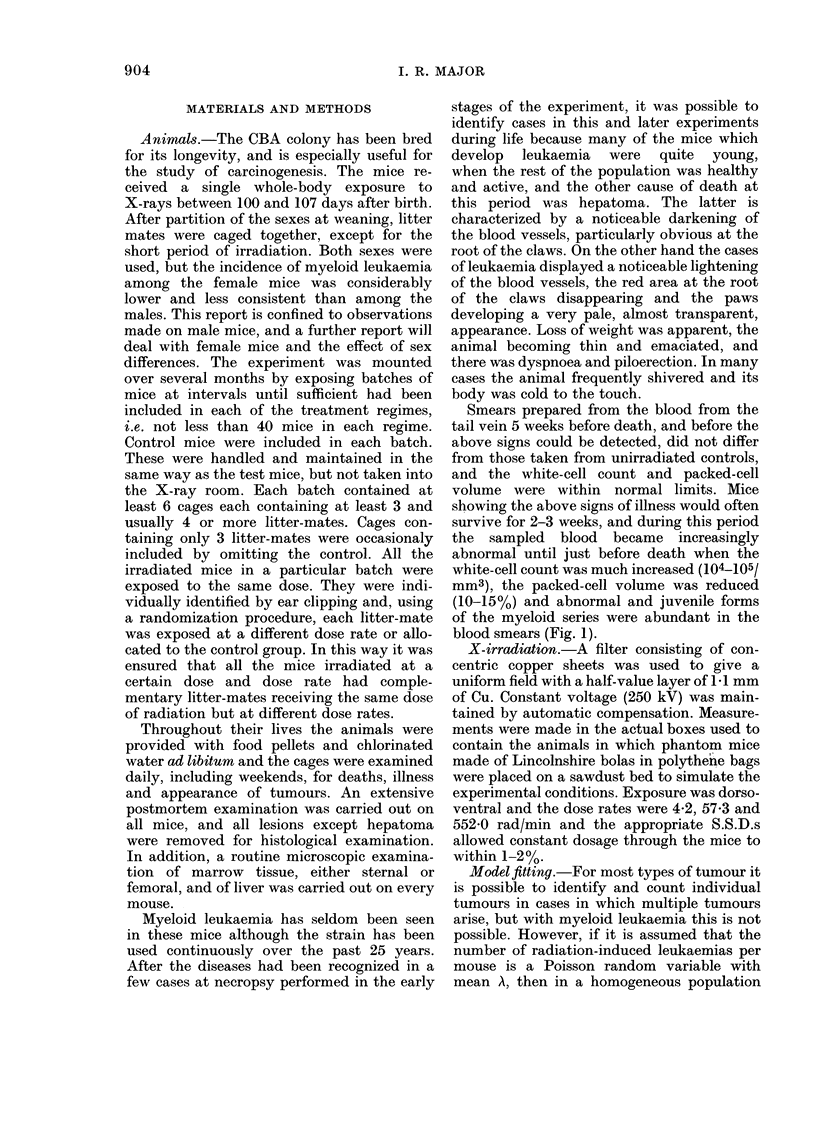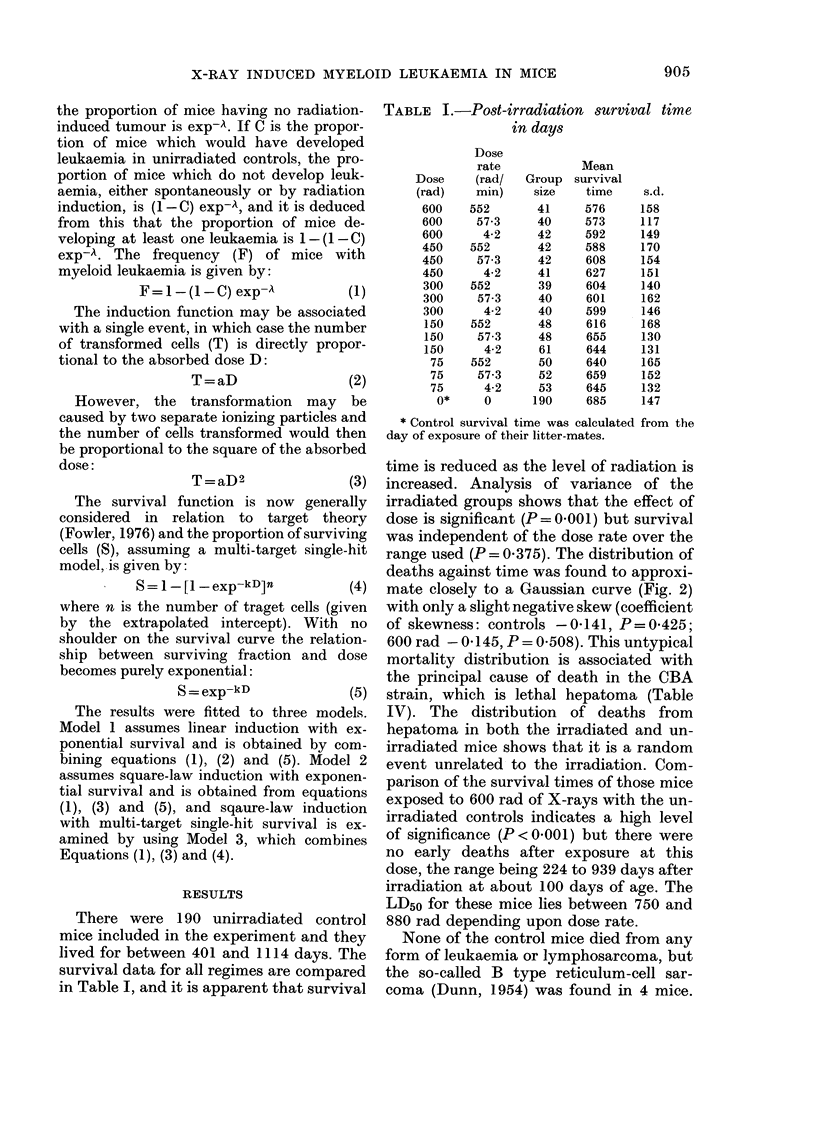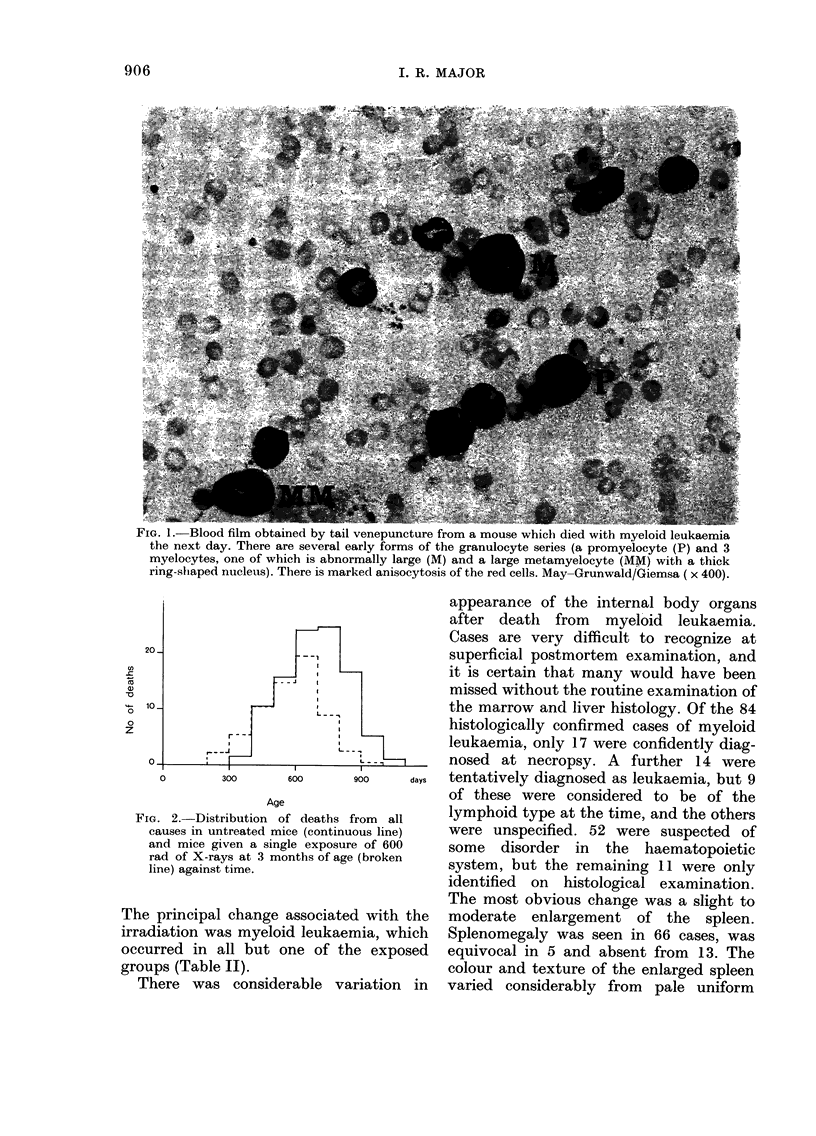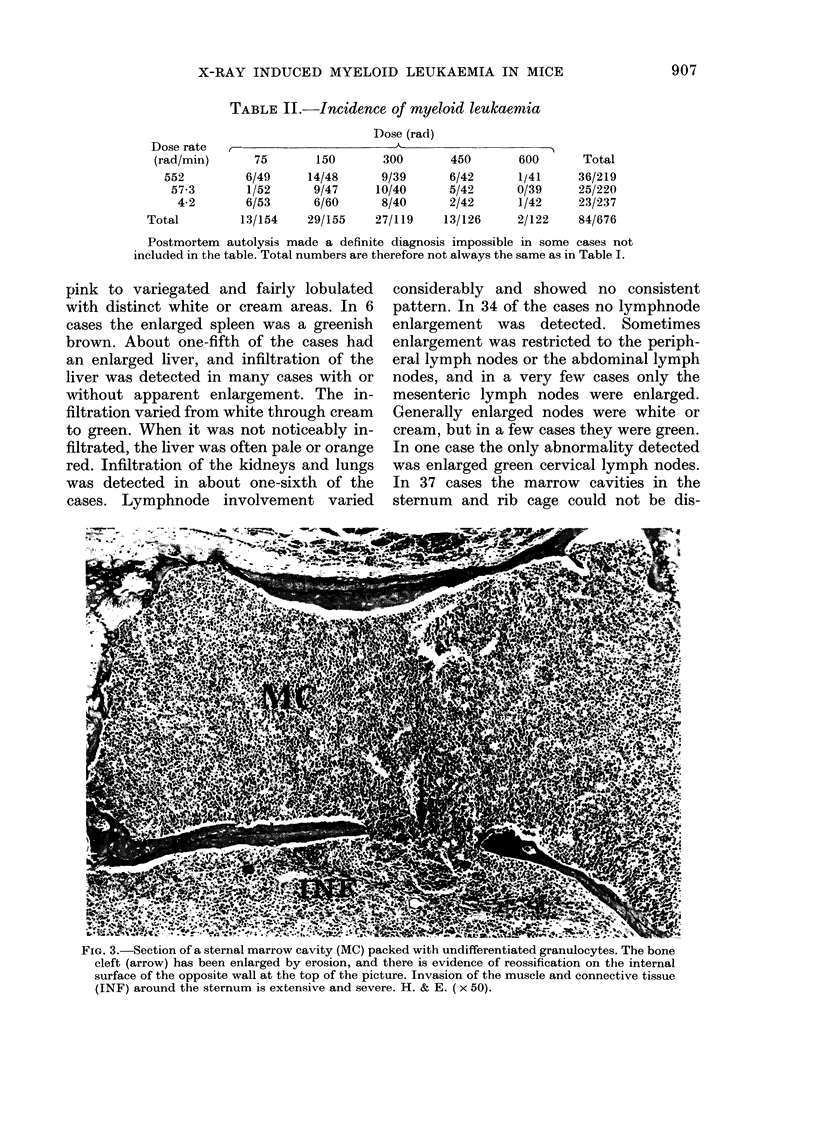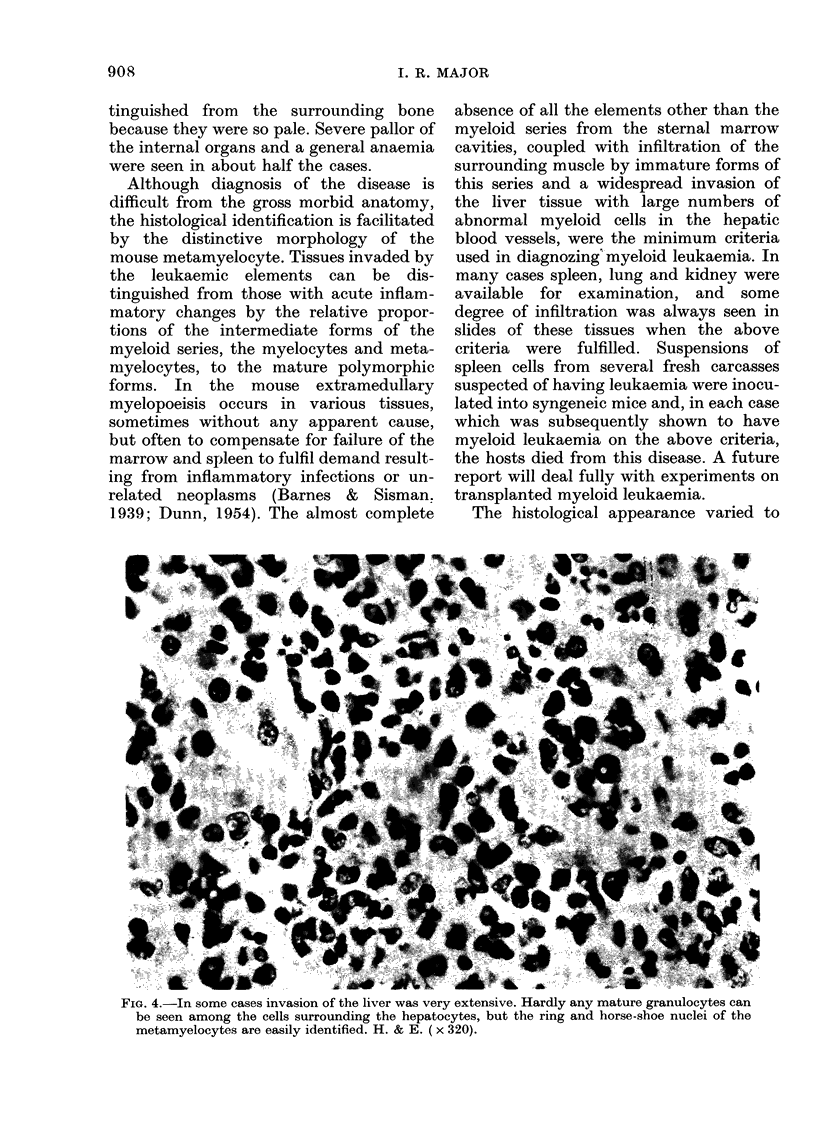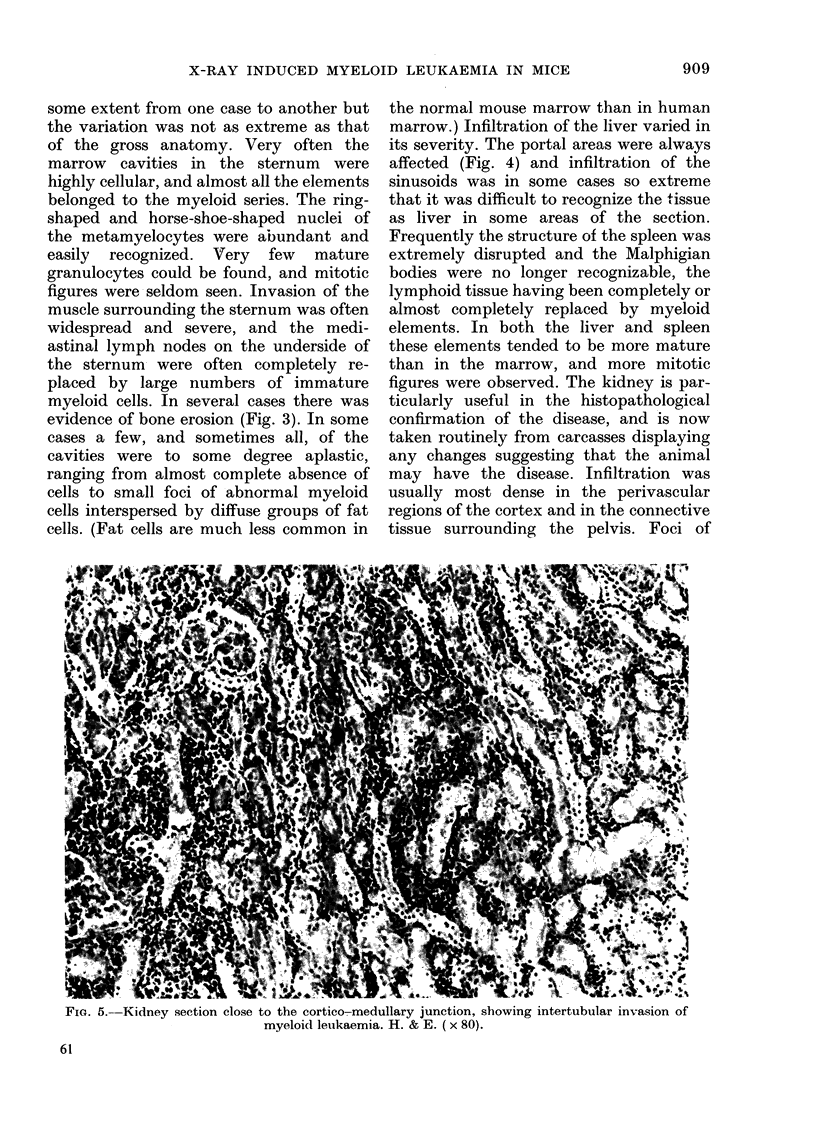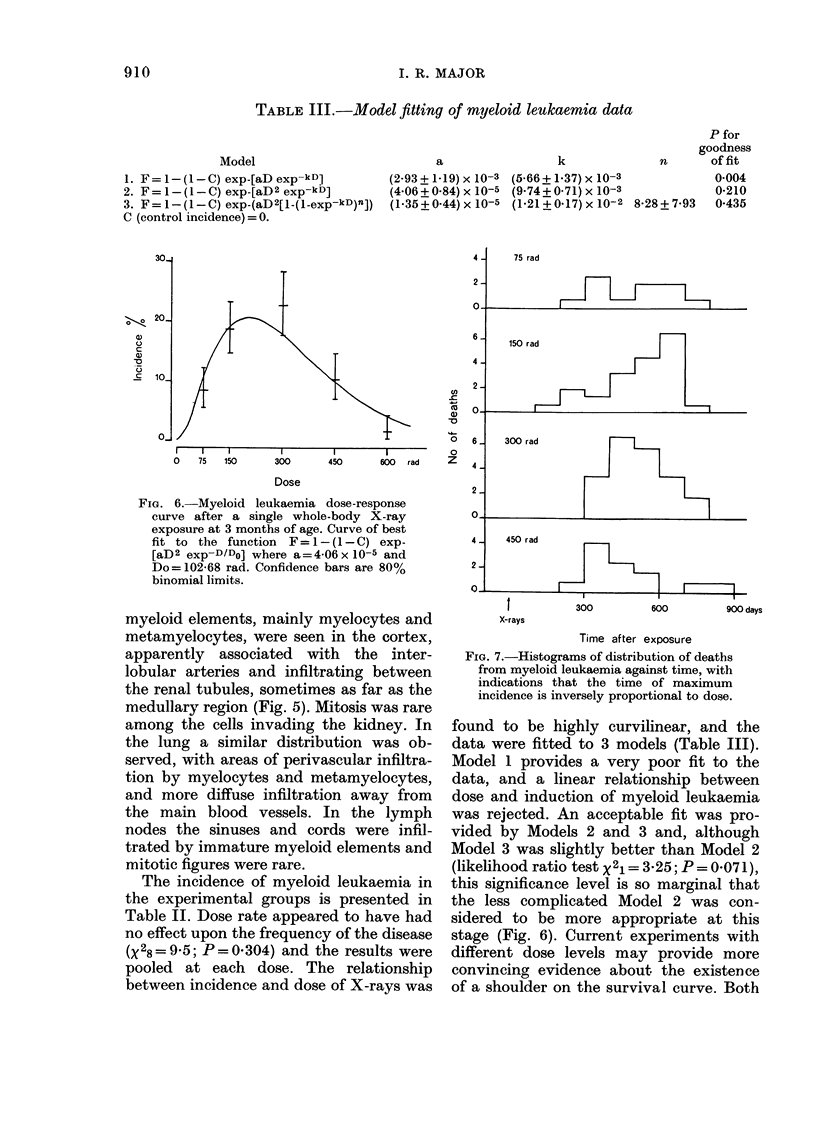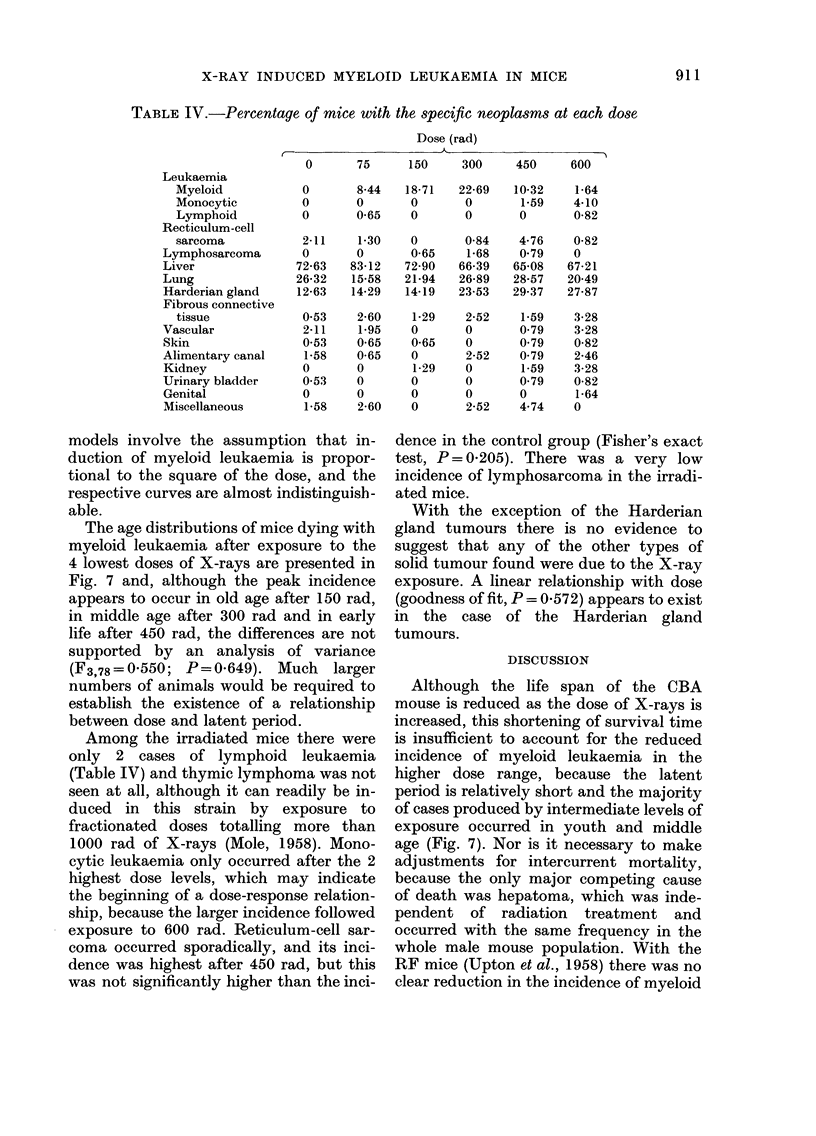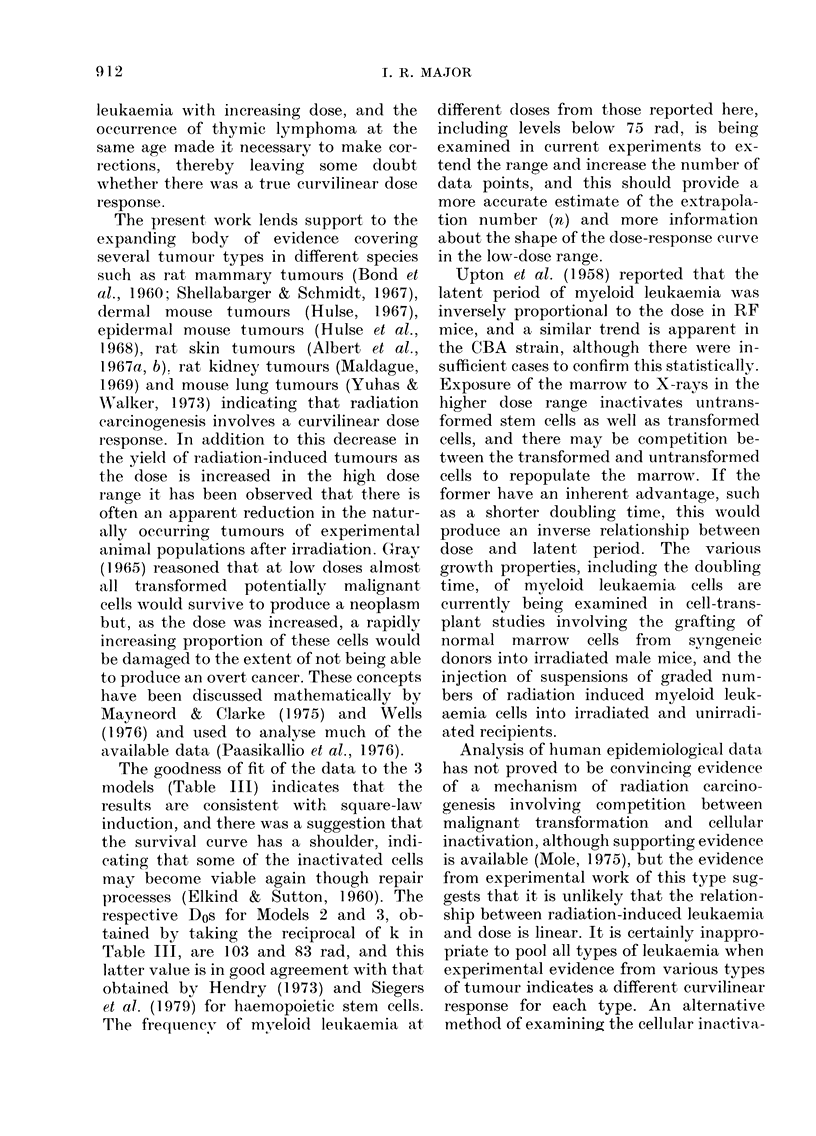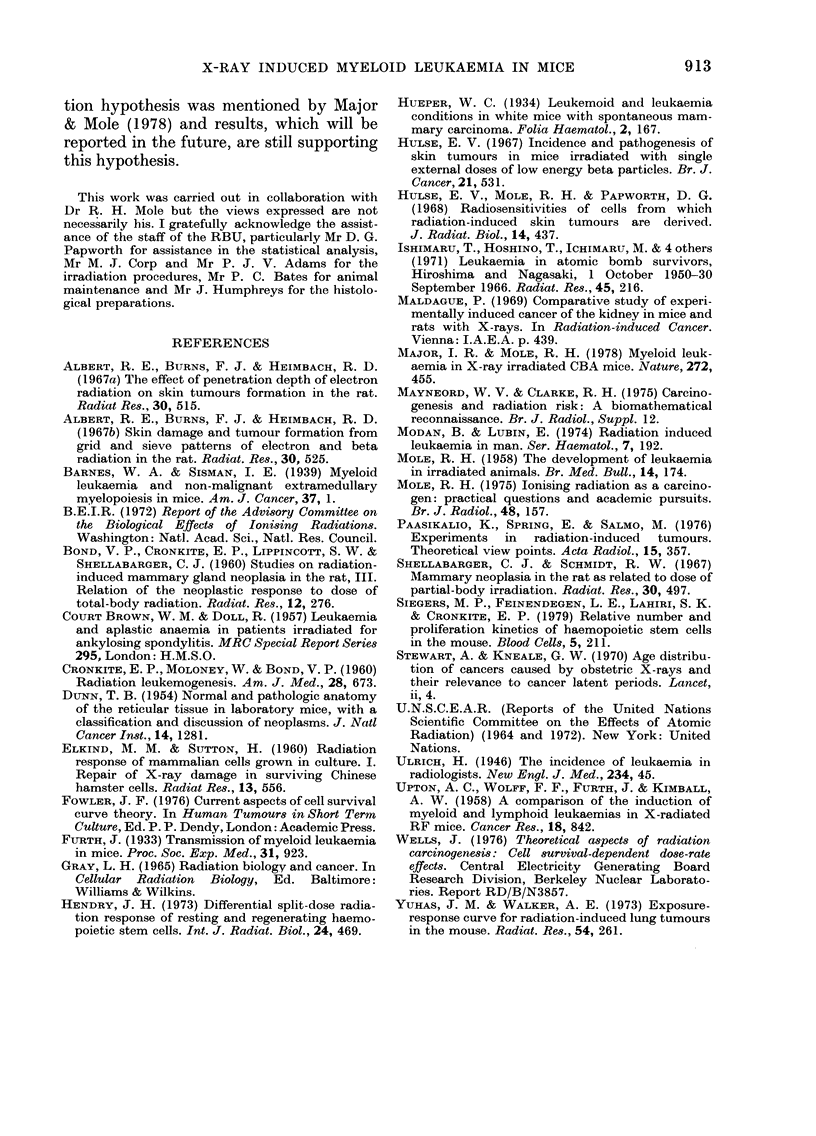# Induction of myeloid leukaemia by whole-body single exposure of CBA male mice to x-rays.

**DOI:** 10.1038/bjc.1979.284

**Published:** 1979-12

**Authors:** I. R. Major

## Abstract

**Images:**


Br. J. Cancer (1979) 40, 903

### INDUCTION OF MYELOID LEUKAEMIA BY WHOLE-BODY SINGLE EXPOSURE OF CBA MALE MICE TO X-RAYS

I. R. MAJOR

*From the MRC Radiobiology Unit, Harwell, Didcot, Oxon*

Received 4 May 1979 Accepted 23 July 1979

**Summary.—A curvilinear dose response of myeloid leukaemia induction in the CBA male mouse was obtained after single whole-body X-irradiation at about 3 months of age. This strain has a very low spontaneous incidence of the disease and no cases were found in the unirradiated controls. The incidence was independent of dose rate over the range used (4.2–552 rad/min). Diagnosis required histopathological examination of various body tissues, the gross anatomical changes being easily confused with other haemopoietic disorders, but a few cases were recognized in life from blood samples. Curve-fitting to various models based on theories of radiocarcinogenic mechanism is described.**

A CONSIDERABLE amount of information is available on cancer induction by ionizing radiation in man (B.E.I.R., 1972; U.N.S.C.E.A.R., 1972). Sufficient data were available in 1960 for Cronkite *et al.* to report that for high-level single-dose exposure of man the incidence of leukaemia of all types was approximately linear with dose, and in 1974 Modan & Lubin stated that it was generally accepted that the relationship between ionizing radiation and human leukaemia is linear and threshold free. Myeloid leukaemia is known to be a hazard of exposure to ionizing radiation of human populations, 58% of the leukaemia cases in the Japanese atom-bomb survivors being of the myeloid type (Ishimaru *et al.*, 1971). It was also preponderant among American radiologists (Ulrich, 1946; U.N.S.C.E.A.R., 1964), patients treated with X-rays for ankylosing spondylitis (Court Brown & Doll, 1957) and children irradiated as foetuses (Stewart & Kneale, 1970).

Different strains of mice show strikingly different responses when exposed to radiation, but the major type of haemopoietic neoplasm is thymic lymphoma, which is rare in humans. In most strains of mice only the lymphoid types of neoplasm have developed and much experimental work has been carried out on these.

There have been sporadic reports of radiation-induced myeloid leukaemia in mice over several years (*e.g.* Furth, 1933; Hueper, 1934) but no systematic investigation was carried out until the early 1950s when Upton and his colleagues investigated the effect of various types of ionizing radiation on the RF strain of mouse at Oak Ridge. This strain has a spontaneous incidence of about 4% of myeloid leukaemia which was increased by single whole-body exposure and produced what appeared to be a non-linear dose-response relationship.

It seems to have been assumed that myeloid leukaemia could only be induced by ionizing radiation in strains of mice which are particularly susceptible to the disease, *i.e.* with a fairly high spontaneous incidence. In this study it is shown that the disease can readily be induced in the Harwell CBA strain of mice in which *all* types of natural leukaemia are rare, and the system provides an excellent model in which to study the leukaemogenic mechanism in detail.

### MATERIALS AND METHODS

*Animals*.—The CBA colony has been bred for its longevity, and is especially useful for the study of carcinogenesis. The mice received a single whole-body exposure to X-rays between 100 and 107 days after birth. After partition of the sexes at weaning, litter mates were caged together, except for the short period of irradiation. Both sexes were used, but the incidence of myeloid leukaemia among the female mice was considerably lower and less consistent than among the males. This report is confined to observations made on male mice, and a further report will deal with female mice and the effect of sex differences. The experiment was mounted over several months by exposing batches of mice at intervals until sufficient had been included in each of the treatment regimes, *i.e.* not less than 40 mice in each regime. Control mice were included in each batch. These were handled and maintained in the same way as the test mice, but not taken into the X-ray room. Each batch contained at least 6 cages each containing at least 3 and usually 4 or more litter-mates. Cages containing only 3 litter-mates were occasionaly included by omitting the control. All the irradiated mice in a particular batch were exposed to the same dose. They were individually identified by ear clipping and, using a randomization procedure, each litter-mate was exposed at a different dose rate or allocated to the control group. In this way it was ensured that all the mice irradiated at a certain dose and dose rate had complementary litter-mates receiving the same dose of radiation but at different dose rates.

Throughout their lives the animals were provided with food pellets and chlorinated water *ad libitum* and the cages were examined daily, including weekends, for deaths, illness and appearance of tumours. An extensive postmortem examination was carried out on all mice, and all lesions except hepatoma were removed for histological examination. In addition, a routine microscopic examination of marrow tissue, either sternal or femoral, and of liver was carried out on every mouse.

Myeloid leukaemia has seldom been seen in these mice although the strain has been used continuously over the past 25 years. After the diseases had been recognized in a few cases at necropsy performed in the early stages of the experiment, it was possible to identify cases in this and later experiments during life because many of the mice which develop leukaemia were quite young, when the rest of the population was healthy and active, and the other cause of death at this period was hepatoma. The latter is characterized by a noticeable darkening of the blood vessels, particularly obvious at the root of the claws. On the other hand the cases of leukaemia displayed a noticeable lightening of the blood vessels, the red area at the root of the claws disappearing and the paws developing a very pale, almost transparent, appearance. Loss of weight was apparent, the animal becoming thin and emaciated, and there was dyspnoea and piloerection. In many cases the animal frequently shivered and its body was cold to the touch.

Smears prepared from the blood from the tail vein 5 weeks before death, and before the above signs could be detected, did not differ from those taken from unirradiated controls, and the white-cell count and packed-cell volume were within normal limits. Mice showing the above signs of illness would often survive for 2–3 weeks, and during this period the sampled blood became increasingly abnormal until just before death when the white-cell count was much increased ($10^4$–$10^5$/mm$^3$), the packed-cell volume was reduced (10–15%) and abnormal and juvenile forms of the myeloid series were abundant in the blood smears (Fig. 1).

*X-irradiation*.—A filter consisting of concentric copper sheets was used to give a uniform field with a half-value layer of 1·1 mm of Cu. Constant voltage (250 kV) was maintained by automatic compensation. Measurements were made in the actual boxes used to contain the animals in which phantom mice made of Lincolnshire bolas in polythene bags were placed on a sawdust bed to simulate the experimental conditions. Exposure was dorsoventral and the dose rates were 4·2, 57·3 and 552·0 rad/min and the appropriate S.S.D.s allowed constant dosage through the mice to within 1–2%.

*Model fitting*.—For most types of tumour it is possible to identify and count individual tumours in cases in which multiple tumours arise, but with myeloid leukaemia this is not possible. However, if it is assumed that the number of radiation-induced leukaemias per mouse is a Poisson random variable with mean $\lambda$, then in a homogeneous population the proportion of mice having no radiation-induced tumour is $\exp^{-\lambda}$. If C is the proportion of mice which would have developed leukaemia in unirradiated controls, the proportion of mice which do not develop leukaemia, either spontaneously or by radiation induction, is $(1-C)\exp^{-\lambda}$, and it is deduced from this that the proportion of mice developing at least one leukaemia is $1-(1-C)\exp^{-\lambda}$. The frequency (F) of mice with myeloid leukaemia is given by:

$$F = 1-(1-C)\exp^{-\lambda} \quad (1)$$

The induction function may be associated with a single event, in which case the number of transformed cells (T) is directly proportional to the absorbed dose D:

$$T = aD \quad (2)$$

However, the transformation may be caused by two separate ionizing particles and the number of cells transformed would then be proportional to the square of the absorbed dose:

$$T = aD^2 \quad (3)$$

The survival function is now generally considered in relation to target theory (Fowler, 1976) and the proportion of surviving cells (S), assuming a multi-target single-hit model, is given by:

$$S = 1-[1-\exp^{-kD}]^n \quad (4)$$

where $n$ is the number of traget cells (given by the extrapolated intercept). With no shoulder on the survival curve the relationship between surviving fraction and dose becomes purely exponential:

$$S = \exp^{-kD} \quad (5)$$

The results were fitted to three models. Model 1 assumes linear induction with exponential survival and is obtained by combining equations (1), (2) and (5). Model 2 assumes square-law induction with exponential survival and is obtained from equations (1), (3) and (5), and sqaure-law induction with multi-target single-hit survival is examined by using Model 3, which combines Equations (1), (3) and (4).

### RESULTS

There were 190 unirradiated control mice included in the experiment and they lived for between 401 and 1114 days. The survival data for all regimes are compared in Table I, and it is apparent that survival time is reduced as the level of radiation is increased. Analysis of variance of the irradiated groups shows that the effect of dose is significant ($P = 0.001$) but survival was independent of the dose rate over the range used ($P = 0.375$). The distribution of deaths against time was found to approximate closely to a Gaussian curve (Fig. 2) with only a slight negative skew (coefficient of skewness: controls $-0.141$, $P = 0.425$; 600 rad $-0.145$, $P = 0.508$). This untypical mortality distribution is associated with the principal cause of death in the CBA strain, which is lethal hepatoma (Table IV). The distribution of deaths from hepatoma in both the irradiated and unirradiated mice shows that it is a random event unrelated to the irradiation. Comparison of the survival times of those mice exposed to 600 rad of X-rays with the unirradiated controls indicates a high level of significance ($P < 0.001$) but there were no early deaths after exposure at this dose, the range being 224 to 939 days after irradiation at about 100 days of age. The $LD_{50}$ for these mice lies between 750 and 880 rad depending upon dose rate.

None of the control mice died from any form of leukaemia or lymphosarcoma, but the so-called B type reticulum-cell sarcoma (Dunn, 1954) was found in 4 mice.

TABLE I.—*Post-irradiation survival time in days*

| Dose (rad) | Dose rate (rad/min) | Group size | Mean survival time | s.d. |
|---|---|---|---|---|
| 600 | 552 | 41 | 576 | 158 |
| 600 | 57·3 | 40 | 573 | 117 |
| 600 | 4·2 | 42 | 592 | 149 |
| 450 | 552 | 42 | 588 | 170 |
| 450 | 57·3 | 42 | 608 | 154 |
| 450 | 4·2 | 41 | 627 | 151 |
| 300 | 552 | 39 | 604 | 140 |
| 300 | 57·3 | 40 | 601 | 162 |
| 300 | 4·2 | 40 | 599 | 146 |
| 150 | 552 | 48 | 616 | 168 |
| 150 | 57·3 | 48 | 655 | 130 |
| 150 | 4·2 | 61 | 644 | 131 |
| 75 | 552 | 50 | 640 | 165 |
| 75 | 57·3 | 52 | 659 | 152 |
| 75 | 4·2 | 53 | 645 | 132 |
| 0* | 0 | 190 | 685 | 147 |

* Control survival time was calculated from the day of exposure of their litter-mates.

FIG. 1.—Blood film obtained by tail venepuncture from a mouse which died with myeloid leukaemia the next day. There are several early forms of the granulocyte series (a promyelocyte (P) and 3 myelocytes, one of which is abnormally large (M) and a large metamyelocyte (MM) with a thick ring-shaped nucleus). There is marked anisocytosis of the red cells. May–Grunwald/Giemsa ( × 400).

FIG. 2.—Distribution of deaths from all causes in untreated mice (continuous line) and mice given a single exposure of 600 rad of X-rays at 3 months of age (broken line) against time.

The principal change associated with the irradiation was myeloid leukaemia, which occurred in all but one of the exposed groups (Table II).

There was considerable variation in appearance of the internal body organs after death from myeloid leukaemia. Cases are very difficult to recognize at superficial postmortem examination, and it is certain that many would have been missed without the routine examination of the marrow and liver histology. Of the 84 histologically confirmed cases of myeloid leukaemia, only 17 were confidently diagnosed at necropsy. A further 14 were tentatively diagnosed as leukaemia, but 9 of these were considered to be of the lymphoid type at the time, and the others were unspecified. 52 were suspected of some disorder in the haematopoietic system, but the remaining 11 were only identified on histological examination. The most obvious change was a slight to moderate enlargement of the spleen. Splenomegaly was seen in 66 cases, was equivocal in 5 and absent from 13. The colour and texture of the enlarged spleen varied considerably from pale uniform

TABLE II.—*Incidence of myeloid leukaemia*

| Dose rate (rad/min) | Dose (rad) 75 | 150 | 300 | 450 | 600 | Total |
|---|---|---|---|---|---|---|
| 552 | 6/49 | 14/48 | 9/39 | 6/42 | 1/41 | 36/219 |
| 57·3 | 1/52 | 9/47 | 10/40 | 5/42 | 0/39 | 25/220 |
| 4·2 | 6/53 | 6/60 | 8/40 | 2/42 | 1/42 | 23/237 |
| Total | 13/154 | 29/155 | 27/119 | 13/126 | 2/122 | 84/676 |

Postmortem autolysis made a definite diagnosis impossible in some cases not included in the table. Total numbers are therefore not always the same as in Table I.

pink to variegated and fairly lobulated with distinct white or cream areas. In 6 cases the enlarged spleen was a greenish brown. About one-fifth of the cases had an enlarged liver, and infiltration of the liver was detected in many cases with or without apparent enlargement. The infiltration varied from white through cream to green. When it was not noticeably infiltrated, the liver was often pale or orange red. Infiltration of the kidneys and lungs was detected in about one-sixth of the cases. Lymphnode involvement varied considerably and showed no consistent pattern. In 34 of the cases no lymphnode enlargement was detected. Sometimes enlargement was restricted to the peripheral lymph nodes or the abdominal lymph nodes, and in a very few cases only the mesenteric lymph nodes were enlarged. Generally enlarged nodes were white or cream, but in a few cases they were green. In one case the only abnormality detected was enlarged green cervical lymph nodes. In 37 cases the marrow cavities in the sternum and rib cage could not be dis-

FIG. 3.—Section of a sternal marrow cavity (MC) packed with undifferentiated granulocytes. The bone cleft (arrow) has been enlarged by erosion, and there is evidence of reossification on the internal surface of the opposite wall at the top of the picture. Invasion of the muscle and connective tissue (INF) around the sternum is extensive and severe. H. & E. (×50).

tinguished from the surrounding bone because they were so pale. Severe pallor of the internal organs and a general anaemia were seen in about half the cases.

Although diagnosis of the disease is difficult from the gross morbid anatomy, the histological identification is facilitated by the distinctive morphology of the mouse metamyelocyte. Tissues invaded by the leukaemic elements can be distinguished from those with acute inflammatory changes by the relative proportions of the intermediate forms of the myeloid series, the myelocytes and metamyelocytes, to the mature polymorphic forms. In the mouse extramedullary myelopoeisis occurs in various tissues, sometimes without any apparent cause, but often to compensate for failure of the marrow and spleen to fulfil demand resulting from inflammatory infections or unrelated neoplasms (Barnes & Sisman, 1939; Dunn, 1954). The almost complete absence of all the elements other than the myeloid series from the sternal marrow cavities, coupled with infiltration of the surrounding muscle by immature forms of this series and a widespread invasion of the liver tissue with large numbers of abnormal myeloid cells in the hepatic blood vessels, were the minimum criteria used in diagnozing myeloid leukaemia. In many cases spleen, lung and kidney were available for examination, and some degree of infiltration was always seen in slides of these tissues when the above criteria were fulfilled. Suspensions of spleen cells from several fresh carcasses suspected of having leukaemia were inoculated into syngeneic mice and, in each case which was subsequently shown to have myeloid leukaemia on the above criteria, the hosts died from this disease. A future report will deal fully with experiments on transplanted myeloid leukaemia.

The histological appearance varied to

Fig. 4.—In some cases invasion of the liver was very extensive. Hardly any mature granulocytes can be seen among the cells surrounding the hepatocytes, but the ring and horse-shoe nuclei of the metamyelocytes are easily identified. H. & E. ( × 320).

some extent from one case to another but the variation was not as extreme as that of the gross anatomy. Very often the marrow cavities in the sternum were highly cellular, and almost all the elements belonged to the myeloid series. The ring-shaped and horse-shoe-shaped nuclei of the metamyelocytes were abundant and easily recognized. Very few mature granulocytes could be found, and mitotic figures were seldom seen. Invasion of the muscle surrounding the sternum was often widespread and severe, and the mediastinal lymph nodes on the underside of the sternum were often completely replaced by large numbers of immature myeloid cells. In several cases there was evidence of bone erosion (Fig. 3). In some cases a few, and sometimes all, of the cavities were to some degree aplastic, ranging from almost complete absence of cells to small foci of abnormal myeloid cells interspersed by diffuse groups of fat cells. (Fat cells are much less common in the normal mouse marrow than in human marrow.) Infiltration of the liver varied in its severity. The portal areas were always affected (Fig. 4) and infiltration of the sinusoids was in some cases so extreme that it was difficult to recognize the tissue as liver in some areas of the section. Frequently the structure of the spleen was extremely disrupted and the Malphigian bodies were no longer recognizable, the lymphoid tissue having been completely or almost completely replaced by myeloid elements. In both the liver and spleen these elements tended to be more mature than in the marrow, and more mitotic figures were observed. The kidney is particularly useful in the histopathological confirmation of the disease, and is now taken routinely from carcasses displaying any changes suggesting that the animal may have the disease. Infiltration was usually most dense in the perivascular regions of the cortex and in the connective tissue surrounding the pelvis. Foci of

FIG. 5.—Kidney section close to the cortico-medullary junction, showing intertubular invasion of myeloid leukaemia. H. & E. (× 80).

TABLE III.—*Model fitting of myeloid leukaemia data*

| Model | a | k | *n* | *P* for goodness of fit |
|---|---|---|---|---|
| 1. $F=1-(1-C)\ \exp\text{-}[aD\ \exp^{-kD}]$ | $(2{\cdot}93\pm1{\cdot}19)\times10^{-3}$ | $(5{\cdot}66\pm1{\cdot}37)\times10^{-3}$ | | 0·004 |
| 2. $F=1-(1-C)\ \exp\text{-}[aD^2\ \exp^{-kD}]$ | $(4{\cdot}06\pm0{\cdot}84)\times10^{-5}$ | $(9{\cdot}74\pm0{\cdot}71)\times10^{-3}$ | | 0·210 |
| 3. $F=1-(1-C)\ \exp\text{-}(aD^2[1\text{-}(1\text{-}\exp^{-kD})^n])$ | $(1{\cdot}35\pm0{\cdot}44)\times10^{-5}$ | $(1{\cdot}21\pm0{\cdot}17)\times10^{-2}$ | $8{\cdot}28\pm7{\cdot}93$ | 0·435 |

C (control incidence) = 0.

FIG. 6.—Myeloid leukaemia dose-response curve after a single whole-body X-ray exposure at 3 months of age. Curve of best fit to the function $F=1-(1-C)$ exp-$[aD^2\ \exp^{-D/D_0}]$ where $a=4{\cdot}06\times10^{-5}$ and $D_0=102{\cdot}68$ rad. Confidence bars are 80% binomial limits.

FIG. 7.—Histograms of distribution of deaths from myeloid leukaemia against time, with indications that the time of maximum incidence is inversely proportional to dose.

myeloid elements, mainly myelocytes and metamyelocytes, were seen in the cortex, apparently associated with the interlobular arteries and infiltrating between the renal tubules, sometimes as far as the medullary region (Fig. 5). Mitosis was rare among the cells invading the kidney. In the lung a similar distribution was observed, with areas of perivascular infiltration by myelocytes and metamyelocytes, and more diffuse infiltration away from the main blood vessels. In the lymph nodes the sinuses and cords were infiltrated by immature myeloid elements and mitotic figures were rare.

The incidence of myeloid leukaemia in the experimental groups is presented in Table II. Dose rate appeared to have had no effect upon the frequency of the disease ($\chi^2_8=9{\cdot}5$; $P=0{\cdot}304$) and the results were pooled at each dose. The relationship between incidence and dose of X-rays was found to be highly curvilinear, and the data were fitted to 3 models (Table III). Model 1 provides a very poor fit to the data, and a linear relationship between dose and induction of myeloid leukaemia was rejected. An acceptable fit was provided by Models 2 and 3 and, although Model 3 was slightly better than Model 2 (likelihood ratio test $\chi^2_1=3{\cdot}25$; $P=0{\cdot}071$), this significance level is so marginal that the less complicated Model 2 was considered to be more appropriate at this stage (Fig. 6). Current experiments with different dose levels may provide more convincing evidence about the existence of a shoulder on the survival curve. Both

TABLE IV.—*Percentage of mice with the specific neoplasms at each dose*

| | Dose (rad) | | | | | |
|---|---|---|---|---|---|---|
| | 0 | 75 | 150 | 300 | 450 | 600 |
| Leukaemia | | | | | | |
| Myeloid | 0 | 8·44 | 18·71 | 22·69 | 10·32 | 1·64 |
| Monocytic | 0 | 0 | 0 | 0 | 1·59 | 4·10 |
| Lymphoid | 0 | 0·65 | 0 | 0 | 0 | 0·82 |
| Recticulum-cell sarcoma | 2·11 | 1·30 | 0 | 0·84 | 4·76 | 0·82 |
| Lymphosarcoma | 0 | 0 | 0·65 | 1·68 | 0·79 | 0 |
| Liver | 72·63 | 83·12 | 72·90 | 66·39 | 65·08 | 67·21 |
| Lung | 26·32 | 15·58 | 21·94 | 26·89 | 28·57 | 20·49 |
| Harderian gland | 12·63 | 14·29 | 14·19 | 23·53 | 29·37 | 27·87 |
| Fibrous connective tissue | 0·53 | 2·60 | 1·29 | 2·52 | 1·59 | 3·28 |
| Vascular | 2·11 | 1·95 | 0 | 0 | 0·79 | 3·28 |
| Skin | 0·53 | 0·65 | 0·65 | 0 | 0·79 | 0·82 |
| Alimentary canal | 1·58 | 0·65 | 0 | 2·52 | 0·79 | 2·46 |
| Kidney | 0 | 0 | 1·29 | 0 | 1·59 | 3·28 |
| Urinary bladder | 0·53 | 0 | 0 | 0 | 0·79 | 0·82 |
| Genital | 0 | 0 | 0 | 0 | 0 | 1·64 |
| Miscellaneous | 1·58 | 2·60 | 0 | 2·52 | 4·74 | 0 |

models involve the assumption that induction of myeloid leukaemia is proportional to the square of the dose, and the respective curves are almost indistinguishable.

The age distributions of mice dying with myeloid leukaemia after exposure to the 4 lowest doses of X-rays are presented in Fig. 7 and, although the peak incidence appears to occur in old age after 150 rad, in middle age after 300 rad and in early life after 450 rad, the differences are not supported by an analysis of variance ($F_{3,78} = 0·550$; $P = 0·649$). Much larger numbers of animals would be required to establish the existence of a relationship between dose and latent period.

Among the irradiated mice there were only 2 cases of lymphoid leukaemia (Table IV) and thymic lymphoma was not seen at all, although it can readily be induced in this strain by exposure to fractionated doses totalling more than 1000 rad of X-rays (Mole, 1958). Monocytic leukaemia only occurred after the 2 highest dose levels, which may indicate the beginning of a dose-response relationship, because the larger incidence followed exposure to 600 rad. Reticulum-cell sarcoma occurred sporadically, and its incidence was highest after 450 rad, but this was not significantly higher than the incidence in the control group (Fisher's exact test, $P = 0·205$). There was a very low incidence of lymphosarcoma in the irradiated mice.

With the exception of the Harderian gland tumours there is no evidence to suggest that any of the other types of solid tumour found were due to the X-ray exposure. A linear relationship with dose (goodness of fit, $P = 0·572$) appears to exist in the case of the Harderian gland tumours.

### DISCUSSION

Although the life span of the CBA mouse is reduced as the dose of X-rays is increased, this shortening of survival time is insufficient to account for the reduced incidence of myeloid leukaemia in the higher dose range, because the latent period is relatively short and the majority of cases produced by intermediate levels of exposure occurred in youth and middle age (Fig. 7). Nor is it necessary to make adjustments for intercurrent mortality, because the only major competing cause of death was hepatoma, which was independent of radiation treatment and occurred with the same frequency in the whole male mouse population. With the RF mice (Upton *et al.*, 1958) there was no clear reduction in the incidence of myeloid leukaemia with increasing dose, and the occurrence of thymic lymphoma at the same age made it necessary to make corrections, thereby leaving some doubt whether there was a true curvilinear dose response.

The present work lends support to the expanding body of evidence covering several tumour types in different species such as rat mammary tumours (Bond *et al.*, 1960; Shellabarger & Schmidt, 1967), dermal mouse tumours (Hulse, 1967), epidermal mouse tumours (Hulse *et al.*, 1968), rat skin tumours (Albert *et al.*, 1967*a*, *b*), rat kidney tumours (Maldague, 1969) and mouse lung tumours (Yuhas & Walker, 1973) indicating that radiation carcinogenesis involves a curvilinear dose response. In addition to this decrease in the yield of radiation-induced tumours as the dose is increased in the high dose range it has been observed that there is often an apparent reduction in the naturally occurring tumours of experimental animal populations after irradiation. Gray (1965) reasoned that at low doses almost all transformed potentially malignant cells would survive to produce a neoplasm but, as the dose was increased, a rapidly increasing proportion of these cells would be damaged to the extent of not being able to produce an overt cancer. These concepts have been discussed mathematically by Mayneord & Clarke (1975) and Wells (1976) and used to analyse much of the available data (Paasikallio *et al.*, 1976).

The goodness of fit of the data to the 3 models (Table III) indicates that the results are consistent with square-law induction, and there was a suggestion that the survival curve has a shoulder, indicating that some of the inactivated cells may become viable again though repair processes (Elkind & Sutton, 1960). The respective $D_0$s for Models 2 and 3, obtained by taking the reciprocal of k in Table III, are 103 and 83 rad, and this latter value is in good agreement with that obtained by Hendry (1973) and Siegers *et al.* (1979) for haemopoietic stem cells. The frequency of myeloid leukaemia at different doses from those reported here, including levels below 75 rad, is being examined in current experiments to extend the range and increase the number of data points, and this should provide a more accurate estimate of the extrapolation number ($n$) and more information about the shape of the dose-response curve in the low-dose range.

Upton *et al.* (1958) reported that the latent period of myeloid leukaemia was inversely proportional to the dose in RF mice, and a similar trend is apparent in the CBA strain, although there were insufficient cases to confirm this statistically. Exposure of the marrow to X-rays in the higher dose range inactivates untransformed stem cells as well as transformed cells, and there may be competition between the transformed and untransformed cells to repopulate the marrow. If the former have an inherent advantage, such as a shorter doubling time, this would produce an inverse relationship between dose and latent period. The various growth properties, including the doubling time, of myeloid leukaemia cells are currently being examined in cell-transplant studies involving the grafting of normal marrow cells from syngeneic donors into irradiated male mice, and the injection of suspensions of graded numbers of radiation induced myeloid leukaemia cells into irradiated and unirradiated recipients.

Analysis of human epidemiological data has not proved to be convincing evidence of a mechanism of radiation carcinogenesis involving competition between malignant transformation and cellular inactivation, although supporting evidence is available (Mole, 1975), but the evidence from experimental work of this type suggests that it is unlikely that the relationship between radiation-induced leukaemia and dose is linear. It is certainly inappropriate to pool all types of leukaemia when experimental evidence from various types of tumour indicates a different curvilinear response for each type. An alternative method of examining the cellular inactivation hypothesis was mentioned by Major & Mole (1978) and results, which will be reported in the future, are still supporting this hypothesis.

This work was carried out in collaboration with Dr R. H. Mole but the views expressed are not necessarily his. I gratefully acknowledge the assistance of the staff of the RBU, particularly Mr D. G. Papworth for assistance in the statistical analysis, Mr M. J. Corp and Mr P. J. V. Adams for the irradiation procedures, Mr P. C. Bates for animal maintenance and Mr J. Humphreys for the histological preparations.

### REFERENCES

Albert, R. E., Burns, F. J. & Heimbach, R. D. (1967*a*) The effect of penetration depth of electron radiation on skin tumours formation in the rat. *Radiat Res.*, **30**, 515.

Albert, R. E., Burns, F. J. & Heimbach, R. D. (1967*b*) Skin damage and tumour formation from grid and sieve patterns of electron and beta radiation in the rat. *Radiat. Res.*, **30**, 525.

Barnes, W. A. & Sisman, I. E. (1939) Myeloid leukaemia and non-malignant extramedullary myelopoiesis in mice. *Am. J. Cancer*, **37**, 1.

B.E.I.R. (1972) *Report of the Advisory Committee on the Biological Effects of Ionising Radiations.* Washington: Natl. Acad. Sci., Natl. Res. Council.

Bond, V. P., Cronkite, E. P., Lippincott, S. W. & Shellabarger, C. J. (1960) Studies on radiation-induced mammary gland neoplasia in the rat, III. Relation of the neoplastic response to dose of total-body radiation. *Radiat. Res.*, **12**, 276.

Court Brown, W. M. & Doll, R. (1957) Leukaemia and aplastic anaemia in patients irradiated for ankylosing spondylitis. *MRC Special Report Series* **295**, London: H.M.S.O.

Cronkite, E. P., Moloney, W. & Bond, V. P. (1960) Radiation leukemogenesis. *Am. J. Med.*, **28**, 673.

Dunn, T. B. (1954) Normal and pathologic anatomy of the reticular tissue in laboratory mice, with a classification and discussion of neoplasms. *J. Natl Cancer Inst.*, **14**, 1281.

Elkind, M. M. & Sutton, H. (1960) Radiation response of mammalian cells grown in culture. I. Repair of X-ray damage in surviving Chinese hamster cells. *Radiat Res.*, **13**, 556.

Fowler, J. F. (1976) Current aspects of cell survival curve theory. In *Human Tumours in Short Term Culture*, Ed. P. P. Dendy, London: Academic Press.

Furth, J. (1933) Transmission of myeloid leukaemia in mice. *Proc. Soc. Exp. Med.*, **31**, 923.

Gray, L. H. (1965) Radiation biology and cancer. In *Cellular Radiation Biology*, Ed. Baltimore: Williams & Wilkins.

Hendry, J. H. (1973) Differential split-dose radiation response of resting and regenerating haemopoietic stem cells. *Int. J. Radiat. Biol.*, **24**, 469.

Hueper, W. C. (1934) Leukemoid and leukaemia conditions in white mice with spontaneous mammary carcinoma. *Folia Haematol.*, **2**, 167.

Hulse, E. V. (1967) Incidence and pathogenesis of skin tumours in mice irradiated with single external doses of low energy beta particles. *Br. J. Cancer*, **21**, 531.

Hulse, E. V., Mole, R. H. & Papworth, D. G. (1968) Radiosensitivities of cells from which radiation-induced skin tumours are derived. *J. Radiat. Biol.*, **14**, 437.

Ishimaru, T., Hoshino, T., Ichimaru, M. & 4 others (1971) Leukaemia in atomic bomb survivors, Hiroshima and Nagasaki, 1 October 1950–30 September 1966. *Radiat. Res.*, **45**, 216.

Maldague, P. (1969) Comparative study of experimentally induced cancer of the kidney in mice and rats with X-rays. In *Radiation-induced Cancer.* Vienna: I.A.E.A. p. 439.

Major, I. R. & Mole, R. H. (1978) Myeloid leukaemia in X-ray irradiated CBA mice. *Nature*, **272**, 455.

Mayneord, W. V. & Clarke, R. H. (1975) Carcinogenesis and radiation risk: A biomathematical reconnaissance. *Br. J. Radiol., Suppl.* 12.

Modan, B. & Lubin, E. (1974) Radiation induced leukaemia in man. *Ser. Haematol.*, **7**, 192.

Mole, R. H. (1958) The development of leukaemia in irradiated animals. *Br. Med. Bull.*, **14**, 174.

Mole, R. H. (1975) Ionising radiation as a carcinogen: practical questions and academic pursuits. *Br. J. Radiol.*, **48**, 157.

Paasikalio, K., Spring, E. & Salmo, M. (1976) Experiments in radiation-induced tumours. Theoretical view points. *Acta Radiol.*, **15**, 357.

Shellabarger, C. J. & Schmidt, R. W. (1967) Mammary neoplasia in the rat as related to dose of partial-body irradiation. *Radiat. Res.*, **30**, 497.

Siegers, M. P., Feinendegen, L. E., Lahiri, S. K. & Cronkite, E. P. (1979) Relative number and proliferation kinetics of haemopoietic stem cells in the mouse. *Blood Cells*, **5**, 211.

Stewart, A. & Kneale, G. W. (1970) Age distribution of cancers caused by obstetric X-rays and their relevance to cancer latent periods. *Lancet*, ii, 4.

U.N.S.C.E.A.R. (Reports of the United Nations Scientific Committee on the Effects of Atomic Radiation) (1964 and 1972). New York: United Nations.

Ulrich, H. (1946) The incidence of leukaemia in radiologists. *New Engl. J. Med.*, **234**, 45.

Upton, A. C., Wolff, F. F., Furth, J. & Kimball, A. W. (1958) A comparison of the induction of myeloid and lymphoid leukaemias in X-radiated RF mice. *Cancer Res.*, **18**, 842.

Wells, J. (1976) *Theoretical aspects of radiation carcinogenesis: Cell survival-dependent dose-rate effects.* Central Electricity Generating Board Research Division, Berkeley Nuclear Laboratories. Report RD/B/N3857.

Yuhas, J. M. & Walker, A. E. (1973) Exposure-response curve for radiation-induced lung tumours in the mouse. *Radiat. Res.*, **54**, 261.